# Inverse dose-response to gp140 YU2 foldon trimer formulated with aluminum phosphate and ISCOMATRIX® adjuvants

**DOI:** 10.1186/1742-4690-9-S2-P14

**Published:** 2012-09-13

**Authors:** A Wilson, R Powell, S Hoffenberg, A Carpov, H Arendt, J DeStefano, MJ Caulfield

**Affiliations:** 1 International AIDS Vaccine Initiative, Brooklyn, NY, USA

## Background

Conventional vaccine approaches based on delivery of HIV-1 envelope (Env) proteins or peptides derived from Env sequences have failed to generate broadly neutralizing antibodies (bNAbs) to the virus. Even with large doses (200 ug) of adjuvanted gp120 proteins administered multiple times to human volunteers, the subsequent antibody response boosts only moderately with each succeeding vaccination, and titers drop precipitously thereafter. We hypothesized that the usual practice of administering a moderate to high antigen doses may be counter productive to the goal of eliciting durable, high-affinity antibody responses.

## Methods

We conducted a rabbit immunogenicity study in which we compared the anti-gp120 antibody response of rabbits immunized with low (1 ug), medium (10 ug) and high (100 ug) quantities of gp140 YU2 foldon trimer (FT) formulated with aluminum phosphate (alum) alone or in combination with ISCOMATRIX® adjuvant. In addition, we used a more protracted vaccination regimen by administering the vaccine at 0, 8, and 24 week time points.

## Results

Antibody responses elicited by the different YU2 FT vaccination regimens were quantified by ELISA against JRCSF gp120 protein after vaccination showing weak responses after the first two vaccine doses. However, after 3 doses, the responses to vaccines co-formulated with both ISCOMATRIX® and alum were markedly higher than the corresponding responses to the antigen formulated with alum only. Interestingly, at 4 weeks post-dose 3, there was a reverse dose response effect, with the 1 ug dose group having higher titers than the 10 and 100 ug groups. At 12 weeks post-dose 3, the antibody GMTs were 28,078 (1 ug), 9,681 (10 ug) and 7,253 (100 ug).

## Conclusion

The results showing that the low dose group maintained a response ~4 fold higher than the high dose group suggests that durability of the antibody response to HIV Env may be a function of antigen dose.

